# HPV DNA Detection and Genotyping in Basal-Like Breast Carcinoma: Insights into a Possible Viral Association

**DOI:** 10.3390/diagnostics16183001

**Published:** 2026-09-16

**Authors:** Angela Santoro, Giuseppe Angelico, Antonio D’Amati, Nicoletta D’Alessandris, Belen Padial Urtueta, Nadine Narducci, Saveria Spadola, Francesca Addante, Gian Franco Zannoni, Antonino Mule

**Affiliations:** 1 Pathology Unit, Department of Woman and Child’s Health and Public Health Sciences, Fondazione Policlinico Universitario Agostino Gemelli IRCCS, Largo A. Gemelli 8, 00168 Rome, Italy; damatiantonio@yahoo.it (A.D.); nicoletta.dalessandris@policlinicogemelli.it (N.D.); belen.padialurtueta@guest.policlinicogemelli.it (B.P.U.); nadine.narducci@libero.it (N.N.); francesca.addante1@gmail.com (F.A.); gianfranco.zannoni@unicatt.it (G.F.Z.); antonino.mule@policlinicogemelli.it (A.M.); 2 Pathology Institute, Catholic University of Sacred Heart, 00168 Rome, Italy; 3 Department of Medicine and Surgery, Kore University of Enna, 94100 Enna, Italy; giuangel86@hotmail.it (G.A.);

**Keywords:** HPV, basal-like carcinoma, immunohistochemistry, breast, p16

## Abstract

**Objectives**: High-risk human papillomavirus (HPV) has been implicated in breast carcinogenesis, but its role remains controversial. Basal-like breast carcinomas (BLCs) share morphological and immunophenotypic features with HPV-related squamous cell cancers. This study aimed to detect HPV DNA in BLCs, perform genotyping, and evaluate p16 expression as a surrogate marker of HPV infection. **Methods**: A total of 36 formalin-fixed, paraffin-embedded basal-like breast carcinomas were studied. Additionally, 10 patients with high-grade (G3) invasive ductal carcinoma of no special type (NOS), of a different molecular subtype, were included as a control group. Immunohistochemistry for p16 was performed, followed by DNA PCR using L1-type specific primers for HPV genotyping in all cases, irrespective of p16 status. Ten high-grade invasive ductal carcinomas served as controls. **Results**: p16 overexpression was observed in 28/36 BLCs (77.8%). Overall, HPV DNA was detected in 11/36 BLCs (30.6%). Among p16-positive BLCs, HPV DNA was detected in 10 cases (35.7%): HPV45 in 4 cases, HPV16 in 4 cases, and HPV18 in 2 cases. One additional BLC that was p16-negative tested positive for HPV16. In the control group, HPV16 DNA was found in 4/5 p16-positive cases. No double infections were identified in the BLC cohort. **Conclusions**: High-risk HPV DNA (types 16, 18, and 45) is present in a substantial subset of p16-positive basal-like breast carcinomas, suggesting a possible pathogenetic role for the virus in these aggressive tumors, although causality remains to be established. However, p16 is not an absolute surrogate for HPV in breast tissue, and molecular confirmation is required. Further studies with E6/E7 mRNA assays are needed to confirm viral transcriptional activity.

## 1. Introduction

It is well established that specific subtypes of human papillomavirus (HPV) are a major cause of human cancers [1,2,3,4]. HPVs are a group of host-specific DNA viruses with remarkable epithelial cell specificity. More than 120 different HPV genotypes have been identified, and almost 45 subtypes isolated from the lower genital tract have been grouped into high-risk and low-risk types based on their potential to induce invasive cancer [5]. Although data regarding HPV prevalence in breast cancer, risk factors, genetic pathways, and molecular pathogenesis remain controversial, recent studies have demonstrated HPV presence in several breast carcinomas by identifying classic koilocytic alterations and suggesting a causal role for the virus in breast carcinogenesis [6,7,8,9,10,11,12,13].

It is well known that HPV16 and HPV18 can promote neoplastic transformation of ductal epithelial cells. Numerous authors have identified oncogenic HPVs, such as HPV16/33/35, in invasive and/or metastatic breast carcinomas [6,7,8,9,10,11,12,13]. Recent large-scale meta-analyses have substantially strengthened this association. A comprehensive 2025 meta-analysis encompassing 49 studies with 4173 breast cancer cases found a pooled HPV DNA prevalence of 23% in breast cancer tissues, with HPV-positive individuals exhibiting a 3.6-fold higher risk of developing breast cancer compared to HPV-negative individuals [14]. Notably, this analysis identified HPV-18 as showing a more consistent correlation with breast cancer than HPV-16, challenging the traditional dominance of HPV-16 in non-genital cancers [14]. Furthermore, a 2025 systematic review on HPV and Epstein–Barr virus (EBV) co-infection reported a 14% pooled prevalence of dual viral presence in breast cancer, with a substantially elevated risk (OR = 5.87, 95% CI: 2.31–14.93), suggesting potential synergistic oncogenic effects [15].

Several studies have emphasized that, in breast parenchyma, the favored host cells for HPV infection are the basal cells of the mammary ducts, which express membranous receptors such as caveolin 1, CD44, CD151, integrin α2β4, and laminin. These are the same molecular markers that identify the basal-like phenotype of breast cancer, which represents 15–20% of all breast cancer subtypes [10,11,12,13,14,16].

Morphologically, basal-like breast carcinomas are high-grade neoplasms composed of sheets of small basaloid cells and large polygonal cells. They typically show pushing margins, extensive areas of comedonecrosis, interstitial lymphoid stroma, and scant desmoplasia [17,18,19].

Although BLCs are defined by gene expression profiling, this technique is not routinely feasible in clinical practice; therefore, an immunohistochemical (IHC) surrogate has been widely adopted for the identification of basal-like tumors [17,18,19].

The most validated IHC definition, requires a tumor to fulfill both of the following criteria: (i) a triple-negative immunophenotype, defined as absence of estrogen receptor (ER) expression (<1% positive tumor cells by IHC), absence of progesterone receptor (PR) expression (<1% positive tumor cells), and absence of HER2/neu overexpression/amplification (IHC 0–1+ or FISH-negative); and (ii) basal marker positivity, defined as expression of at least one of the following basal/myoepithelial markers by IHC—cytokeratin 5/6 (CK5/6), cytokeratin 14 (CK14), cytokeratin 17 (CK17), and/or epidermal growth factor receptor (EGFR), with positivity defined as membranous and/or cytoplasmic staining in ≥10% of tumor cells [17,18,19]. This IHC surrogate panel of four markers (ER, HER1/EGFR, HER2, and CK5/6) demonstrates 100% specificity and 76% sensitivity for identifying basal-like tumors when compared to gene expression profiling as the gold standard [17,18,19].

BLCs preferentially affect young women, have an aggressive clinical behavior, and show morphological overlap with poorly differentiated HPV-related squamous cell carcinomas of other sites, including the uterine cervix, head and neck, esophagus, penis, vulva, anus, and perianal region [2,3,4,16,17,18,19].

The clinical, morphological, and immunophenotypical overlapping characteristics of BLCs and high-grade squamous cell HPV-related cancers raise the hypothesis of a possible viral involvement in a subset of these tumors and suggest that similar molecular pathways, including alterations in the Rb/p16 axis, may be operative in both entities [2,3,4,16,17,18,19]. However, it is important to emphasize that the detection of HPV DNA alone does not establish a causal relationship; viral carcinogenesis requires transcriptional activity of the E6 and E7 oncogenes, and further studies are needed to confirm whether HPV plays a direct pathogenic role in breast carcinogenesis.

Importantly, geographic variation in HPV prevalence within breast cancer has been documented, with significantly higher rates observed in Asian populations compared to other regions [14]. This variability underscores the importance of population-specific studies and suggests potential roles for genetic susceptibility, viral strain distribution, or environmental cofactors.

Methodological differences further complicate cross-study comparisons. HPV detection assays target distinct genomic regions, primarily the L1 capsid gene or the E6/E7 oncogenes [8,9,10,11,12,13,14,15]. L1-targeted PCR may yield false-negative results when viral integration disrupts the L1 region [8,9,10,11,12,13,14,15]. E6/E7-targeted assays better reflect transcriptionally active infections but are type-specific. Nested PCR enhances sensitivity but increases contamination risk [8,9,10,11,12,13,14,15]. These technical variations contribute to the wide range of reported HPV prevalence in breast cancer [8,9,10,11,12,13,14,15]. Consequently, direct comparisons between studies must be interpreted with caution.

In addition to direct HPV DNA detection, immunohistochemical evaluation of p16^(INK4A) has emerged as a valuable surrogate marker for transcriptionally active HPV infection in cervical and oropharyngeal cancers, where diffuse and strong ‘block-type’ immunostaining demonstrates high sensitivity and specificity [1,3,16]. p16 is a cyclin-dependent kinase inhibitor that is markedly overexpressed when the retinoblastoma (Rb) protein is inactivated by the HPV E7 oncoprotein, leading to release of E2F transcription factors and subsequent p16 upregulation through a negative feedback loop [1,3,16]. In breast cancer, however, the utility of p16 as a surrogate marker remains controversial.

In the present study, we aimed to detect HPV DNA in basal-like breast cancer, perform HPV genotyping, and evaluate p16 overexpression in neoplastic cells. Our objectives were to investigate the potential carcinogenic role of HPV in this specific tumor type and to support the development of therapeutic and preventive strategies for this aggressive breast carcinoma subtype.

## 2. Materials and Methods

### 2.1. Study Population

The study group comprised 36 female patients with a histologically and immunohistochemically confirmed diagnosis of triple-negative breast carcinoma with a basal-like immunophenotype (see criteria below). Additionally, 10 patients with high-grade (G3) invasive ductal carcinoma of no special type (NOS), of a different molecular subtype, were included as a control group. Importantly, all control cases were confirmed to be non-basal-like by immunohistochemistry (ER-positive and/or PR-positive, HER2-negative or HER2-positive, with Ki-67 >20%) and lacked expression of basal markers (CK5/6, CK14, CK17).

In total, 36 basal-like carcinomas were retrieved from the Pathology archives at the Pathology Department, Catholic University of the Sacred Heart, Rome. Microscopic evaluation was performed by two breast pathologists, who determined histotype, percentage of in situ and invasive components, Nottingham grade, TNM stage, and presence of lymphovascular invasion [20].

Tumors were defined as triple-negative breast cancers (TNBCs) with a basal-like immunophenotype when they showed: (i) lack of ER, PR, and HER2 expression (‘triple-negative’); and (ii) expression of one or more high-molecular-weight/basal cytokeratins (CK5/6, CK14, or CK17).

### 2.2. Tissues

Archival formalin-fixed, paraffin-embedded (FFPE) breast cancer samples were obtained from the surgical tumor registries of our institution. All samples had been routinely fixed in 10% neutral buffered formalin and embedded in paraffin blocks. For each case, a hematoxylin and eosin-stained slide was reviewed to confirm the presence of tumor and to select a representative block. Corresponding normal parenchyma distant from the tumor was also selected. Tumor cellularity was estimated by two pathologists (A.S. and G.A.) on the H&E-stained reference slide; only cases with a minimum tumor cellularity of 70% were included in the study. Manual microdissection was performed to selectively isolate tumor cell populations from surrounding stroma, inflammatory cells, and benign ductal epithelium, guided by the corresponding H&E-stained reference slide. Tumor-enriched areas were carefully dissected away from stroma, inflammatory cells, in situ components, and benign breast epithelium and then transferred into microcentrifuge tubes for DNA extraction.

### 2.3. Immunohistochemistry

Immunohistochemistry was performed on 4-µm paraffin sections mounted on poly-L-lysine-coated slides using the standard LSAB-HRP technique with a monoclonal antibody against p16 (clone E6H4) on a Benchmark XT autostainer (Ventana Medical Systems Inc., Tucson, AZ, USA). Appropriate positive and negative controls were run for each batch.

Positive controls consisted of sections of known HPV-positive cervical squamous cell carcinoma (previously confirmed by PCR and p16 IHC) and HPV-positive tonsillar squamous cell carcinoma, processed in parallel with each staining batch. Non-neoplastic stromal cells, inflammatory cells, and normal mammary epithelial cells present within each tumor section served as internal negative controls, as these are expected to be p16-negative in the absence of non-specific staining. A known HPV-positive cervical carcinoma section was included in each run as an external positive control to verify staining performance, and a section of the same tumor processed without primary antibody served as an external negative control.

Staining was evaluated by two independent pathologists (A.S. and G.A.) blinded to the clinical and molecular data. p16 immunoreactivity was assessed based on the percentage of positive tumor cells showing both nuclear and cytoplasmic staining. Staining was scored as: 1+ (1–30% positive tumor cells), 2+ (31–60% positive tumor cells), and 3+ (61–90% positive tumor cells). For the purpose of this study, p16 positivity was defined as diffuse and strong (“block-type”) nuclear and cytoplasmic staining involving ≥61% of tumor cells, consistent with the criteria used for HPV-related squamous cell carcinomas [16]. Cases showing only focal, weak, or exclusively cytoplasmic staining were considered negative. In case of disagreement between the two pathologists, consensus was reached by joint re-evaluation using a multi-headed microscope.

### 2.4. DNA Extraction

FFPE sections were deparaffinized with xylene and washed twice with 100% ethanol and once with phosphate-buffered saline. Samples were then incubated overnight at 55 °C in lysis solution containing proteinase K (Qiagen, Venlo, The Netherlands, 20 mg/mL, 50 µL), 1 M Tris-HCl (10 µL), 0.5 M EDTA (2 µL), 10% SDS (100 µL), and distilled water (838 µL). Cross-linking was reversed by adding NaCl to a final concentration of 0.7 M and incubating at 65 °C for 4 h. DNA was purified using the Wizard DNA Clean-Up Kit (Promega, Madison, WI, USA) according to the manufacturer’s protocol. DNA integrity was assessed by PCR amplification of a fragment of the beta-globin gene using primers forward 5′-GAA GAG CCA AGG ACA GGT AC-3′ and reverse 5′-GGA AAA TAG ACC AAT AGG CAG-3′, followed by gel electrophoresis.

### 2.5. PCR Analysis for HPV

All 36 BLCs and the 10 control cases were subjected to PCR analysis. High-risk HPV types 16, 18, 31, 33, and 45 were selected based on their established oncogenic potential and documented prevalence in breast cancer and other HPV-related malignancies [14,21,22]. These genotypes represent the most common high-risk HPV types associated with cervical, anogenital, and oropharyngeal cancers and have been most frequently investigated in breast cancer studies, allowing comparison with the existing literature [14]. High-risk HPV types (16, 18, 31, 33, 45) were detected using a nested PCR assay with L1 consensus primers (GP5+/GP6+) followed by type-specific primers. HPV genotyping was performed by direct sequencing of PCR products. Amplifications were carried out in a Mastercycler gradient thermal cycler (Eppendorf, Hamburg, Germany). Products were resolved on 1% agarose gels, stained with ethidium bromide, and sized against a DNA molecular weight marker. No-template controls (water) and HPV-positive (SiHa cells) and HPV-negative (C33A cells) controls were included in each run. SiHa served as a general control for PCR performance and reagent integrity; for non-HPV16 genotypes, primer specificity has been previously validated, and no-template controls were used to exclude contamination.

The following primer sequences, amplicon sizes, and cycling conditions were utilized:

**DNA Quality Control:**

- Beta-globin PCR performed on all DNA samples to verify integrity;
- Primers: forward 5′-GAAGAGCCAAGGACAGGTAC-3′;
  reverse 5′-GGAAAATAGACCAATAGGCAG-3′;
- Amplicon size: 268 bp;
- Only beta-globin-positive samples were included.

**First-round PCR (GP5+/GP6+ consensus):**

- Reaction volume: 25 µL;
- Template: 100–200 ng DNA;
- Components: 1× PCR buffer, 1.5 mM MgCl_2_, 0.2 mM dNTPs, 0.4 µM each primer, 1.25 U Taq polymerase;
- Cycling: 94 °C/4 min; 40 cycles of 94 °C/30 s, 50 °C/45 s, 72 °C/45 s; 72 °C/7 min;
- Amplicon: 150 bp;
- Visualization: 2% agarose gels.

**Second-round PCR (type-specific):**

- Template: 1 µL first-round product;
- HPV types tested: 16, 18, 31, 33, 45;
- Reaction volume: 25 µL;
- Components: 1× PCR buffer, 2.0 mM MgCl_2_, 0.2 mM dNTPs, 0.4 µM type-specific primers, 1.25 U Taq polymerase;
- Cycling: 94 °C/4 min; 35 cycles of 94 °C/30 s, annealing at 56–58 °C/45 s, 72 °C/45 s; 72 °C/7 min.

**HPV Genotyping (Sequencing):**

- Method: Bidirectional sequencing;
- Kit: BigDye Terminator v3.1;
- Analyzer: ABI Prism 3100;
- Analysis: BLAST (2.16.0+, National Center for Biotechnology Information, Bethesda, MD, USA) comparison with PaVE database references;
- Quality criteria: Phred scores ≥20 for >95% of bases; <2% ambiguous bases;
- Interpretation threshold: ≥98% identity over full amplicon length.

### 2.6. Statistical Analysis

Statistical analysis was performed using Fisher’s exact test to compare HPV positivity between the basal-like and control groups and to evaluate the association between p16 immunohistochemical expression and HPV DNA detection. All tests were two-tailed, and a *p*-value < 0.05 was considered statistically significant. Analyses were performed using GraphPad Prism version 9.0 (GraphPad Software, San Diego, CA, USA).

## 3. Results

### 3.1. Clinicopathological Features

**Figure 1:**
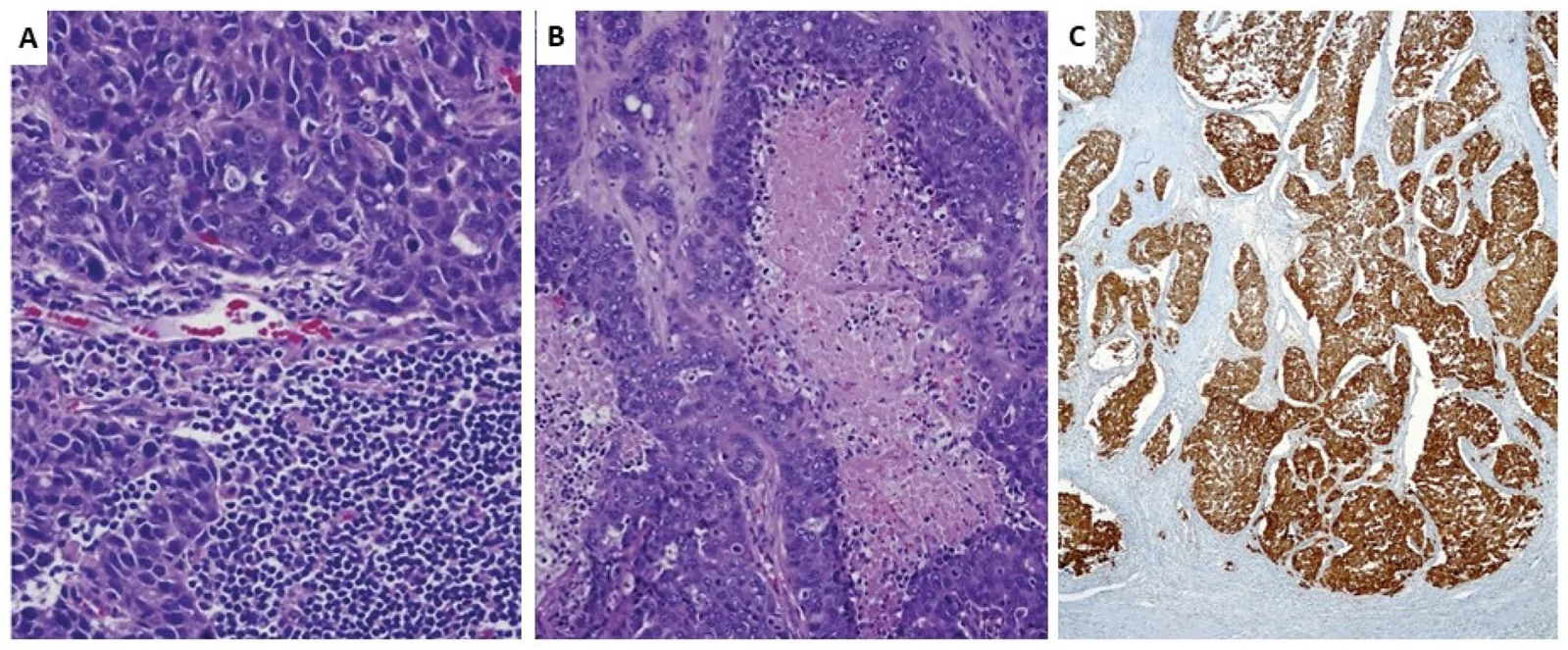
Histopathological and immunohistochemical features of basal-like breast carcinoma. (**A**) High-power view showing sheets of small basaloid cells admixed with large polygonal cells, accompanied by a dense interstitial lymphoid stroma. (**B**) Intraductal comedonecrosis, a characteristic feature of basal-like carcinoma. (**C**) Strong and diffuse p16 immunopositivity in neoplastic cells of a high-grade basal-like carcinoma.

All clinicopathological and immunohistochemical data are analytically summarized in Table 1. A total of 46 women (mean age 51 years, age range 39–61 years) were identified and included in the study. Two study groups were identified: basal-like group and control group. The basal-like group included in the study consisted of 29 invasive ductal carcinomas of no special type (NOS), 6 invasive breast carcinomas with medullary features (formerly designated as medullary carcinoma) and 1 apocrine carcinoma. Morphologically, breast basal-like carcinomas were malignant high-grade neoplasms composed of sheets of small basaloid cells and large polygonal cells (Figure 1A,B). Histologically, BLCs had typical pushing margins, wide areas of comedonecrosis, interstitial lymphoid stroma, and scant desmoplasia.

### 3.2. p16 Immunohistochemistry

Immunohistochemistry for p16 protein as a surrogate marker for HPV infection was performed on all 36 basal-like breast carcinomas and all 10 control cases. In the basal-like group, strong and diffuse immunostaining for p16 was detected in 28 cases (Figure 1C) (77.8%), while 8 cases (22.2%) showed no staining. In the control group, positive p16 staining was detected in five cases (50%).

### 3.3. HPV DNA Detection and Genotyping

Molecular analyses for the detection of HPV infection were performed on all studied tumors with the following results:

Overall, HPV DNA was detected in 11 of 36 basal-like cases (30.6%). In the basal-like group, HPV DNA was detected in 10 of the 28 p16-positive cases (35.7%). Specifically, PCR analysis revealed the presence of HPV45 DNA in 4 cases, HPV16 DNA in 4 cases, and HPV18 DNA in 2 cases. Additionally, HPV16 DNA was detected in 1 of the 8 p16-negative cases (12.5%). In the control group, PCR analysis revealed the presence of HPV16 DNA in 4 of the 5 p16-positive cases (80%).

### 3.4. Statistical Analysis

**Comparison of HPV positivity between groups:**

HPV DNA was detected in 11 of 36 basal-like cases (30.6%) and in 4 of 10 control cases (40.0%). This difference was not statistically significant (*p* = 0.710, Fisher’s exact test).

**Association between p16 expression and HPV DNA positivity:**

In the basal-like group, HPV DNA was detected in 10 of 28 p16-positive cases (35.7%) and in 1 of 8 p16-negative cases (12.5%). This association was not statistically significant (*p* = 0.399, Fisher’s exact test). In the control group, HPV DNA was detected in 4 of 5 p16-positive cases (80.0%) and in 0 of 5 p16-negative cases (0.0%). This association was not statistically significant (*p* = 0.167, Fisher’s exact test).

## 4. Discussion

In this study, we investigated the presence of high-risk human papillomavirus (HPV) DNA in a series of 36 basal-like breast carcinomas and explored the relationship between HPV infection and p16 immunohistochemical expression. Our main finding is that HPV DNA was detected in 11 of 36 BLCs (30.6% overall), including 10 of 28 p16-positive cases (35.7%) and 1 of 8 p16-negative cases (12.5%). Furthermore, one additional BLC that was p16-negative also tested positive for HPV16. These results support the hypothesis that a subgroup of basal-like breast cancers may be driven by HPV, similar to what has been established for cervical, oropharyngeal, and other anogenital squamous cell carcinomas. However, further evidence is needed to establish a causal relationship.

A notable finding in our study was the detection of HPV16 DNA in 4 of the 5 p16-positive control cases (80.0%), compared to 10 of 28 p16-positive basal-like cases (35.7%). Although this difference did not reach statistical significance (*p* = 0.135, Fisher’s exact test), likely due to the small control group size, the high proportion in controls substantially weakens the hypothesis of a specific association between HPV and basal-like breast carcinoma. Several interpretations should be considered. First, HPV DNA detection may not be specific to any particular breast cancer subtype, but rather may occur across different molecular subtypes, possibly reflecting a general susceptibility of breast tissue to viral presence. Second, the control cases were high-grade invasive ductal carcinomas and may share biological features such as high proliferative index, genomic instability, or altered tumor microenvironment, that could favor HPV detection, independent of molecular subtype. Third, it is possible that HPV detection in both groups reflects incidental viral presence rather than a tumor-specific association, particularly in the absence of E6/E7 mRNA confirmation. Collectively, these underscore the need for larger, well-controlled studies incorporating functional assays to determine whether HPV detection in breast tissue has any biological or clinical relevance.

The overall HPV DNA prevalence we observed among p16-positive BLCs (35.7%) is within the wide range reported in the literature (0–86%) for breast cancer [7,8,9,10,11,12,13,14]. This broad variability is largely due to differences in detection methods (PCR vs. next-generation sequencing), sample types (fresh frozen vs. FFPE), and the specific tumor subtypes studied. By focusing exclusively on basal-like tumors, which share morphological and immunophenotypic features with HPV-related squamous cell carcinomas [17,18,19], we likely enriched for cases with a potential viral association. Our prevalence is comparable to the 23% global pooled prevalence recently reported in a large meta-analysis [14] and aligns with findings from Piana et al. (approximately 30% in triple-negative breast cancers) and Corbex et al. (28% in triple-negative/inflammatory tumors) [10,11].

Notably, we detected HPV16 in five cases overall (four p16-positive and one p16-negative), HPV45 in four p16-positive cases, and HPV18 in two p16-positive cases, making HPV16 the most frequently identified genotype in our cohort. This is consistent with the traditional literature identifying HPV16 as the predominant high-risk type in breast cancer [6,7,8,9,10,11,12,13]. At the same time, our finding of HPV45 in a subset of cases (4/11 positive cases, 36%) is noteworthy, as this genotype is less commonly reported in breast cancer but is a well-recognized high-risk type in cervical adenocarcinomas and some head and neck cancers. Interestingly, a recent large meta-analysis reported that HPV18 showed a more consistent association with breast cancer than HPV16 across pooled studies [14]; our cohort did not replicate this pattern, which may reflect differences in geographic distribution, tumor subtype selection (our cohort being restricted to basal-like carcinomas), or sample size. Taken together, the detection of three distinct high-risk genotypes (16, 18, and 45) in our cohort adds to accumulating evidence that breast tissue may be susceptible to a broad range of oncogenic HPV types, and that genotype distribution in breast cancer may not simply mirror that of cervical or other anogenital cancers [14].

The detection of HPV16 in a p16-negative tumor is noteworthy, although HPV detection in breast tissue without corresponding p16 overexpression has been described in the literature. Possible explanations include: (i) viral integration without sufficient p16 upregulation due to alternative RB pathway alterations, (ii) a low viral load below the threshold for p16 induction, or (iii) a false-positive PCR result. Alternatively, it could also indicate that p16 is not an absolutely perfect surrogate for HPV in breast tissue, unlike in the cervix where almost all transcriptionally active HPV infections cause p16 overexpression. This finding is consistent with recent reports from other groups who have identified HPV DNA in p16-negative breast cancers [21], further emphasizing that p16 immunohistochemistry alone is insufficient for HPV detection in breast tissue. Importantly, our HPV detection strategy targeted the L1 region, which may be partially deleted upon viral integration into the host genome, a common event in high-risk HPV-related cancers [1,2,3,4]. Consequently, L1-based PCR may underestimate the true prevalence of HPV in cases where integration has occurred, potentially explaining some discordant p16-positive/HPV-negative results. This limitation underscores the importance of incorporating E6/E7 oncogene assays in future studies to confirm viral presence and transcriptional activity.

An in-depth consideration of HPV detection methodologies is essential for interpreting our findings and comparing them with the literature. The wide variability in reported HPV prevalence in breast cancer (0–86%) is largely attributable to methodological differences rather than true biological variation [1,2,3,4,5]. First, the choice of target genomic region critically influences detection rates. Our study employed L1 consensus primers (GP5+/GP6+), which target the highly conserved capsid gene and allow broad-spectrum HPV detection. However, L1 may be deleted during viral integration into the host genome—a common event in high-risk HPV-related malignancies—potentially leading to false-negative results [1,2,3,4,5]. In contrast, E6/E7-targeted assays are less susceptible to integration-related loss, as these oncogenes are consistently retained in transcriptionally active infections, but they require type-specific primers and do not detect all HPV genotypes in a single reaction [1,2,3,4,5]. Second, nested PCR approaches, such as the one used in our study, enhance analytical sensitivity but also increase the risk of contamination, necessitating rigorous controls and contamination prevention measures [1,2,3,4,5]. Third, differences in DNA extraction methods, FFPE tissue quality and age, and PCR cycling conditions further contribute to inter-study variability [1,2,3,4,5]. Fourth, sequencing-based genotyping, while providing high specificity, may fail to detect low-copy-number infections or mixed genotypes compared to more-sensitive hybridization-based methods [1,2,3,4,5].

In cervical pathology, diffuse and strong p16 immunostaining is considered a highly sensitive and specific surrogate for transforming HPV infection [22,23]. However, its utility in breast cancer is less established. Specifically, our data demonstrate that p16 has poor specificity for HPV DNA positivity in basal-like breast carcinomas. In our series, 28 of 36 BLCs (77.8%) showed p16 positivity, but only 10 of those (35.7%) had detectable HPV DNA. Critically, this means that the majority of p16-positive basal-like cases (18/28, 64.3%) were HPV-negative. This finding strongly suggests that p16 overexpression in basal-like breast carcinomas is largely driven by HPV-independent mechanisms, rather than reflecting HPV infection. Such mechanisms include inactivation of the RB pathway by cyclin D1 amplification, CDKN2A (p16) gene mutation, promoter hypermethylation, or other epigenetic alterations. Indeed, basal-like breast cancers are known to have frequent RB loss and p16 upregulation as a compensatory feedback loop [16]. Furthermore, p16 immunohistochemistry is routinely used as an ancillary diagnostic marker in breast pathology for various purposes, including assessment of proliferation and differential diagnosis, independent of any HPV association [1,2,3,4,5]. This context-dependent diagnostic application further limits its specificity as a surrogate marker for HPV infection in breast tumors. Thus, while p16 immunohistochemistry can help identify a subset of BLCs that may be HPV-related, it is not a standalone surrogate; molecular confirmation is required.

The preferential involvement of basal/myoepithelial cells of the mammary duct by HPV has been proposed by several authors [12,13,14,16]. These cells express integrins, laminin, and caveolin 1 that can serve as receptors for viral entry. The basal-like phenotype, defined by expression of high-molecular-weight cytokeratins (CK5/6, CK14) and EGFR, exactly mirrors the cellular compartment that is susceptible to HPV infection. Therefore, it is biologically plausible that HPV infects these progenitor cells, integrates into the host genome, and drives transformation via E6/E7-mediated p53 and RB degradation. Our finding of HPV DNA in 11 of 36 BLCs (30.6% of the whole cohort) adds to the accumulating evidence that a subset of these aggressive tumors may be virus-associated.

Nevertheless, the exact route of HPV to the breast remains speculative. Proposed mechanisms include hematogenous spread from a genital or oral primary infection, direct inoculation via nipple-areolar manipulation, or transfer by immune cells [12,13]. De Carolis et al. recently showed that HPV DNA can be transferred to breast cancer stromal cells via extracellular vesicles, suggesting a potential mechanism for viral persistence in the tumor microenvironment [13].

Our study does not address viral integration status or transcriptional activity. While the presence of HPV DNA in a subset of BLCs suggests a possible association, p16 positivity in breast tissue does not confirm transcriptionally active HPV infection, as p16 overexpression may result from HPV-independent mechanisms [16]. Therefore, the detection of HPV DNA alone, even when accompanied by p16 expression, does not establish a transforming infection in breast tissue. We acknowledge the limited sample size of this exploratory study, which prevents generalization of our findings. Additional limitations included the absence of E6/E7 mRNA testing, the lack of HPV PCR analysis on corresponding normal breast parenchyma and the use of a single HPV16-positive cell line (SiHa) as the PCR control, which does not provide type-specific validation for HPV18, 31, 33, or 45. Future studies should incorporate a panel of genotype-specific cell line controls to strengthen assay validation. Additionally, the use of an IHC-based definition, rather than molecular gene-expression profiling, represents a limitation. Immunohistochemical surrogates may misclassify a subset of tumors, and the lack of gene-expression data prevents direct comparison with the intrinsic molecular subtypes. Nevertheless, it is important to acknowledge that several well-conducted studies have failed to detect HPV in breast cancer. A 2026 study specifically examining young (under 50), BRCA1/BRCA2-negative breast cancer patients found no detectable high-risk HPV DNA or RNA transcripts in any of the 91 tumor samples analyzed [24], concluding that HPV does not play a role in this specific patient subgroup. These discrepant findings highlight the complexity of the HPV–breast cancer association and suggest that viral involvement may be restricted to particular molecular or clinical subtypes.

## 5. Conclusions

In this study, high-risk HPV DNA (types 16, 18, and 45) was detected in a subset of basal-like breast carcinomas (30.6%), including both p16-positive and p16-negative cases. However, p16 overexpression was also frequently observed in HPV-negative BLCs, indicating that p16 is not a specific surrogate marker for HPV infection in breast tissue. The detection of HPV DNA alone does not establish a causal role for the virus in breast carcinogenesis. However, the impact of HPV in basal-like breast carcinomas remains uncertain and requires further investigation.

Our findings indicate that HPV DNA is present in a subset of basal-like breast carcinomas, but further studies using transcription-based assays (e.g., E6/E7 mRNA detection) and spatially resolved techniques (e.g., in situ hybridization or laser capture microdissection) are needed to determine whether the virus is transcriptionally active within malignant cells and whether it plays a causal, passenger, or incidental role in these tumors.

## Figures and Tables

**Table 1:** Clinicopathological and molecular features of the studied cohort.

Case No.	Histology	Age (yrs)	Tumor Size (cm)	Nodal Status	Stage	LVI	Basal Markers	p16 IHC	HPV DNA	Tumor Cellularity (%)
**Basal-Like Group**										
1	Medullary	47	3.5	N0	IIA	Absent	CK5/6+, CK14+	+	HPV 45	85
2	Medullary	52	4.0	N1	IIB	Present	CK5/6+, CK14+	+	HPV 45	80
3	Medullary	39	2.8	N0	IIA	Absent	CK5/6+, CK14+	+	HPV 45	80
4	Apocrine carcinoma	55	3.2	N1	IIB	Present	CK5/6+, CK14+	+	HPV 45	75
5	IDC, NOS, G3	49	4.5	N2	IIIA	Present	CK5/6+, CK14+, CK17+	+	HPV 18	90
6	IDC, NOS, G3	61	3.8	N0	IIA	Absent	CK5/6+, CK14+	+	HPV 18	85
7	IDC, NOS, G3	44	5.0	N2	IIIA	Present	CK5/6+, CK14+, CK17+	+	HPV 16	90
8	IDC, NOS, G3	51	2.5	N0	IIA	Absent	CK5/6+, CK14+	+	HPV 16	80
9	IDC, NOS, G3	48	4.2	N1	IIB	Present	CK5/6+, CK14+	+	HPV 16	85
10	IDC, NOS, G3	56	3.0	N0	IIA	Absent	CK5/6+, CK14+	+	HPV 16	80
11	IDC, NOS, G3	43	4.8	N2	IIIA	Present	CK5/6+, CK14+, CK17+,	–	HPV 16	90
12	IDC, NOS, G3	50	2.2	N0	IIA	Absent	CK5/6+, CK14+	+	–	75
13	IDC, NOS, G3	47	3.5	N1	IIB	Present	CK5/6+, CK14+	+	–	80
14	IDC, NOS, G3	53	2.8	N0	IIA	Absent	CK5/6+, CK14+	+	–	75
15	Medullary	42	3.0	N0	IIA	Absent	CK5/6+, CK14+	+	–	80
16	IDC, NOS, G3	58	4.5	N1	IIB	Present	CK5/6+, CK14+, CK17+	+	–	85
17	Medullary	45	2.5	N0	IIA	Absent	CK5/6+, CK14+	+	–	80
18	IDC, NOS, G3	51	3.8	N0	IIA	Absent	CK5/6+, CK14+	+	–	75
19	IDC, NOS, G3	49	5.2	N2	IIIA	Present	CK5/6+, CK14+, CK17+	+	–	90
20	IDC, NOS, G3	54	2.0	N0	IIA	Absent	CK5/6+, CK14+	+	–	70
21	IDC, NOS, G3	46	3.5	N1	IIB	Present	CK5/6+, CK14+	+	–	80
22	IDC, NOS, G3	60	4.0	N1	IIB	Present	CK5/6+, CK14+	+	–	85
23	IDC, NOS, G3	41	2.8	N0	IIA	Absent	CK5/6+, CK14+, CK17+	+	–	75
24	IDC, NOS, G3	52	3.2	N0	IIA	Absent	CK5/6+, CK14+	+	–	80
25	IDC, NOS, G3	48	4.5	N2	IIIB	Present	CK5/6+, CK14+, CK17+	+	–	90
26	Medullary	44	2.5	N0	IIA	Absent	CK5/6+, CK14	+	–	80
27	IDC, NOS, G3	57	3.8	N1	IIB	Absent	CK5/6+, CK14+	+	–	85
28	IDC, NOS, G3	50	3.0	N0	IIA	Absent	CK5/6+, CK14+	+	–	75
29	IDC, NOS, G3	53	5.5	N2	IIIA	Present	CK5/6+, CK14+, CK17+,	–	–	90
30	IDC, NOS, G3	46	2.5	N0	IIA	Absent	CK5/6+, CK14+	–	–	70
31	IDC, NOS, G3	59	3.0	N0	IIA	Absent	CK5/6+, CK14+	–	–	75
32	IDC, NOS, G3	42	4.2	N1	IIB	Present	CK5/6+, CK14+	–	–	85
33	IDC, NOS, G3	55	2.8	N0	IIA	Absent	CK5/6+, CK14+	–	–	70
34	IDC, NOS, G3	50	3.5	N0	IIA	Absent	CK5/6+, CK14+	–	–	75
35	IDC, NOS, G3	48	4.0	N1	IIB	Present	CK5/6+, CK14+	–	–	80
36	IDC, NOS, G3	59	3.0	N0	IIA	Absent	CK5/6+, CK14+	–	–	70
**Control Group**										
C1	IDC, NOS, G3	52	3.5	N1	IIB	Present	Negative	+	HPV 16	85
C2	IDC, NOS, G3	48	4.0	N0	IIA	Absent	Negative	+	HPV 16	80
C3	IDC, NOS, G3	55	2.8	N0	IIA	Absent	Negative	+	HPV 16	75
C4	IDC, NOS, G3	41	5.0	N2	IIIA	Present	Negative	+	HPV 16	90
C5	IDC, NOS, G3	60	3.2	N1	IIB	Absent	Negative	+	–	80
C6	IDC, NOS, G3	50	4.5	N0	IIA	Present	Negative	–	–	85
C7	IDC, NOS, G3	44	3.0	N0	IIA	Absent	Negative	–	–	75
C8	IDC, NOS, G3	58	3.8	N1	IIB	Present	Negative	–	–	80
C9	IDC, NOS, G3	50	2.5	N0	IIA	Absent	Negative	–	–	70
C10	IDC, NOS, G3	55	4.2	N0	IIA	Absent	Negative	–	–	75

## Data Availability

Manuscript data are available from the corresponding author upon reasonable request.

## Abbreviations

The following abbreviations are used in this manuscript:

HPV	Human papillomavirus
BLC(s)	Basal-like carcinoma(s)
DNA	Deoxyribonucleic acid
RNA	Ribonucleic acid
mRNA	Messenger ribonucleic acid
PCR	Polymerase chain reaction
FFPE	Formalin-fixed, paraffin-embedded
IHC	Immunohistochemistry
TNBC(s)	Triple-negative breast cancer(s)
ER	Estrogen receptor
PR	Progesterone receptor
HER2	Human epidermal growth factor receptor 2
EGFR	Epidermal growth factor receptor
CK	Cytokeratin
IDC	Invasive ductal carcinoma
NOS	Not otherwise specified
G3	Histological grade 3
TNM	Tumor, Node, Metastasis (staging system)
RB	Retinoblastoma (protein/gene)
CDKN2A	Cyclin-dependent kinase inhibitor 2A
EBV	Epstein–Barr virus
OR	Odds ratio
CI	Confidence interval
LSAB-HRP	Labeled streptavidin–biotin horseradish peroxidase
SDS	Sodium dodecyl sulfate
EDTA	Ethylenediaminetetraacetic acid
BRCA1/BRCA2	Breast cancer gene 1/Breast cancer gene 2
PTEN	Phosphatase and tensin homolog
APOBEC3B	Apolipoprotein B mRNA editing enzyme, catalytic polypeptide-like 3B
miR	MicroRNA
E6/E7	HPV early proteins 6 and 7
GP5+/GP6+	General primer 5+/General primer 6+ (HPV L1 consensus primers)
